# Supplementary material for: Origin of Salt Effects in SN2 Fluorination Using KF Promoted by Ionic Liquids: Quantum Chemical Analysis
Source: Molecules. 2021 Sep 22;26(19):5738. doi: 10.3390/molecules26195738 (PMC8510065; doi:10.3390/molecules26195738)
Supplement: Supplementary file 1 [file molecules-26-05738-s001.zip › molecules-1389064-supplementary.pdf]

Supplementary Information

Origin of Salt Effects in S_N2 Fluorination using KF Promoted by Ionic Liquids: Quantum Chemical Analysis

Young-Ho Oh and Sungyul Lee*

<Case1>

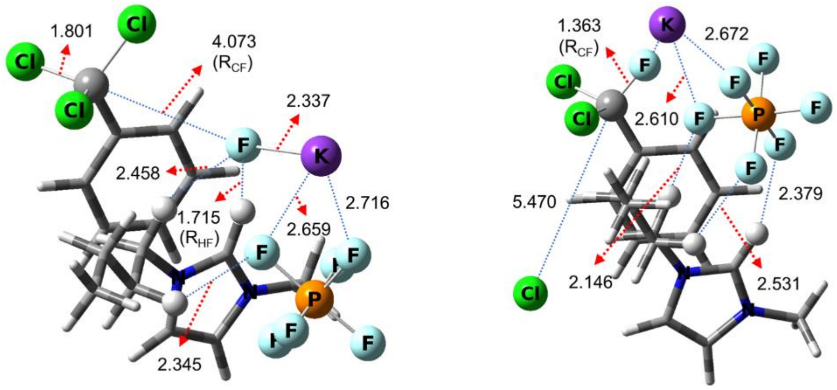

(pre-reaction complex) (post-reaction complex)

<Case2>

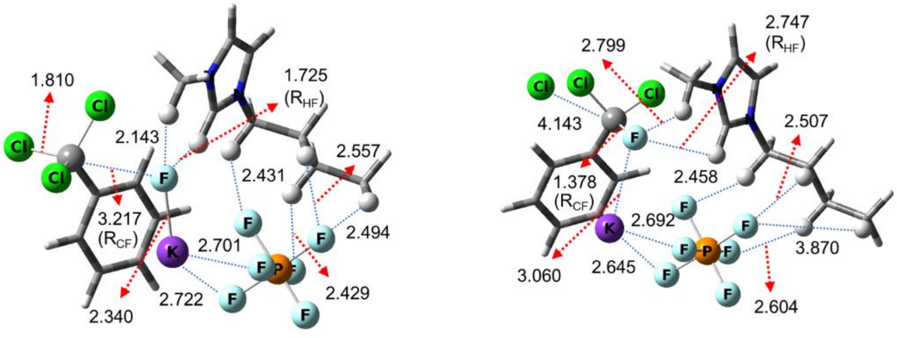

(pre-reaction complex) (post-reaction complex)

Figure 1S. Structures of pre- and post- reaction complexes for S_N2 fluorination in $[bmim]PF_6$ with substrate: KF = 1:1.

<Case1>

<Case2>

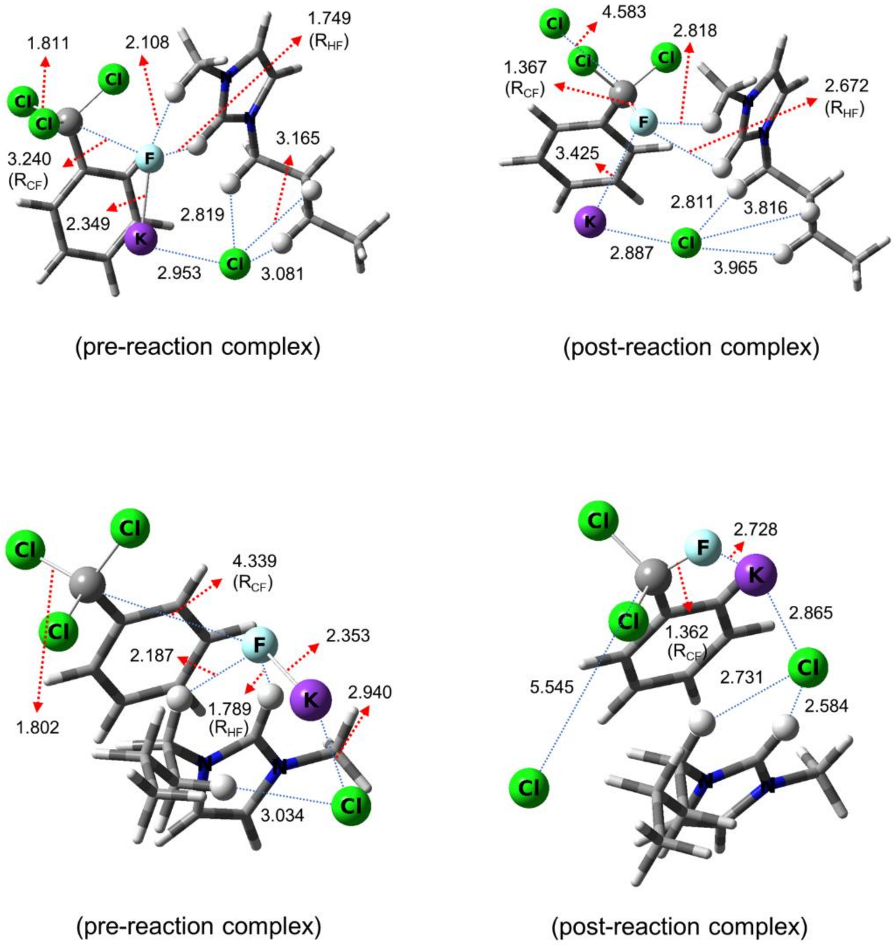

Figure 2S. Structures of pre- and post- reaction complexes Structures of pre- and post- reaction complexes for S_N2 fluorination in [bmim]Cl with substrate: KF = 1:1.

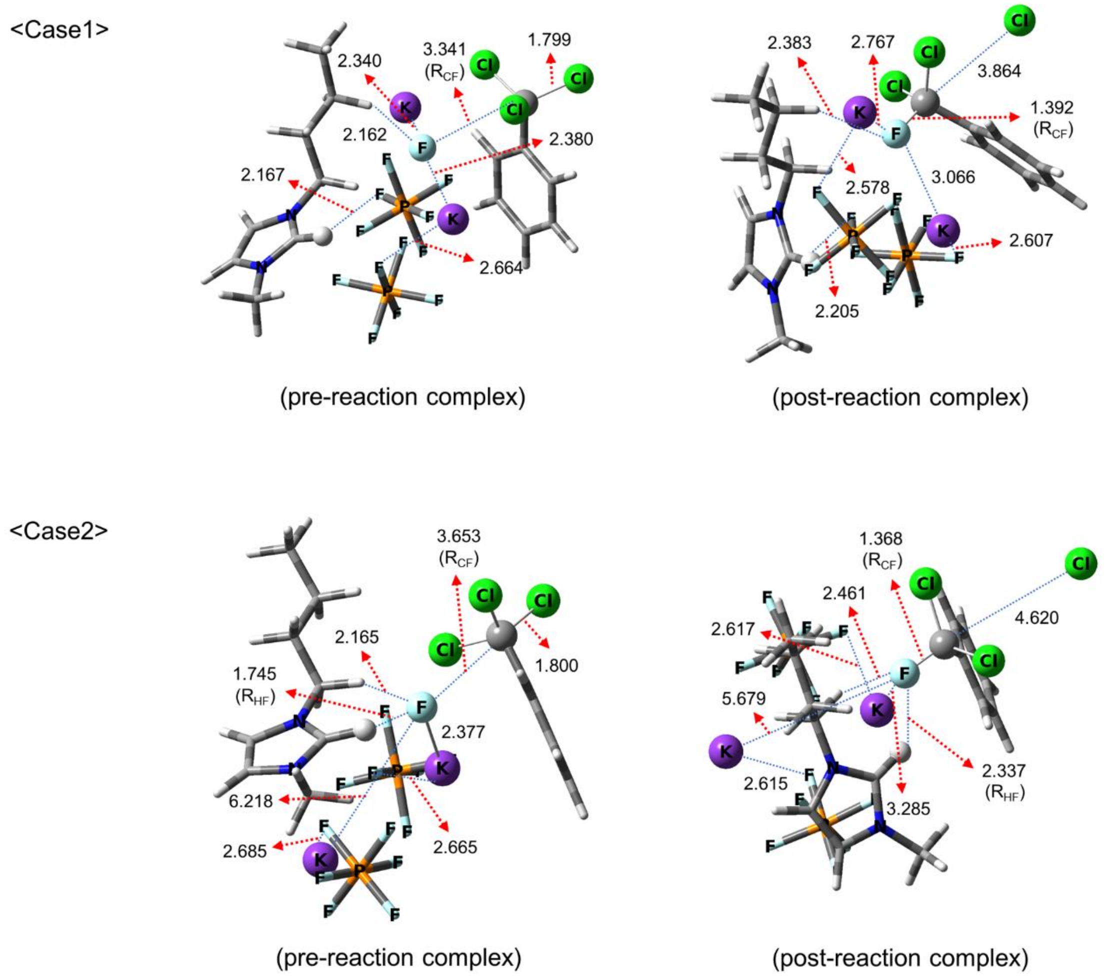

Figure 3S. Structures of pre- and post- reaction complexes for S_N2 fluorination in [bmim]PF_6, with substrate : KF : KPF_6 = 1:1.

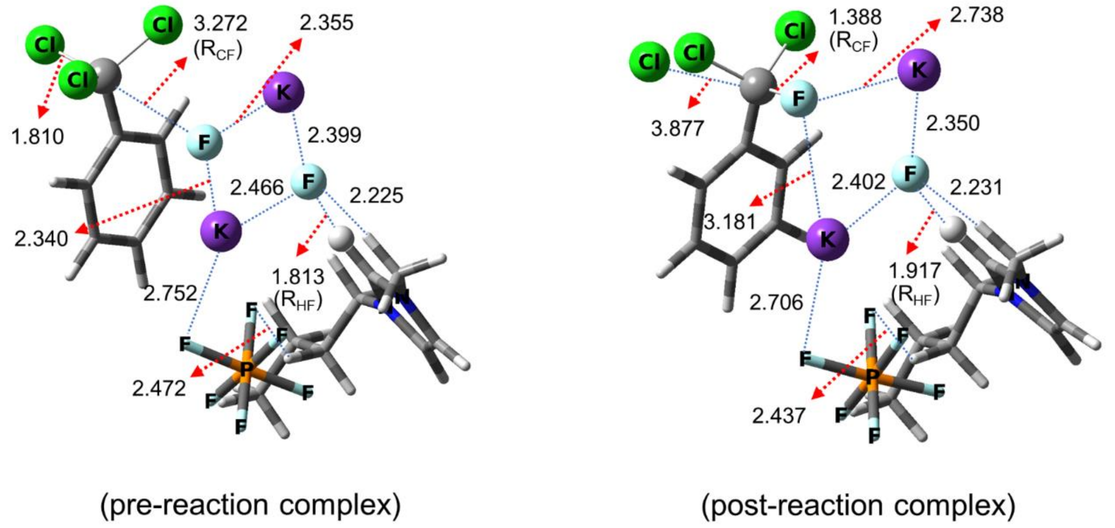

Figure 4S. Structures of pre- and post- reaction complexes for S_N2 fluorination in $[bmim]PF_6$, with substrate : KF = 1:2.

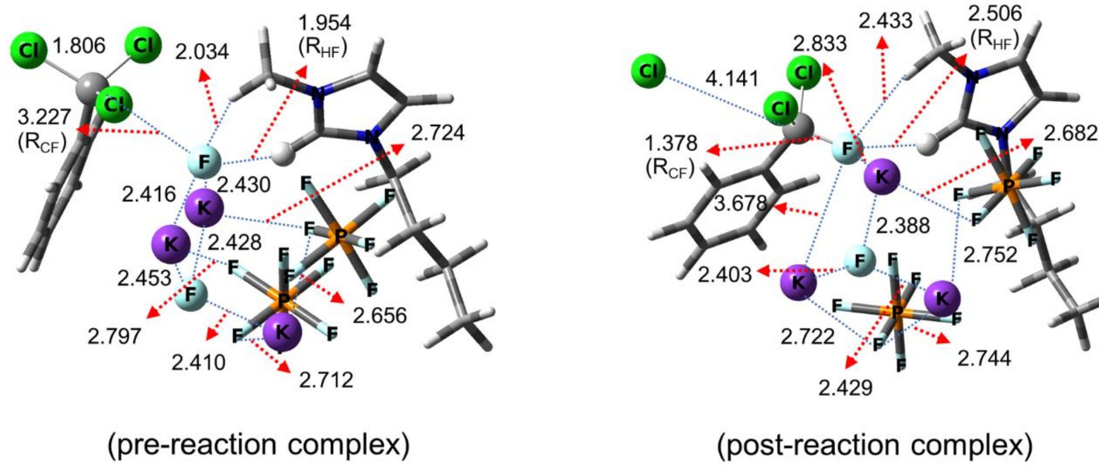

Figure 5S. Structures of pre- and post- reaction complexes for S_N2 fluorination in $[bmim]PF_6$, with substrate : KF : KPF_6 = 1:2:1.

Cartesian coordinates

Figure 1S. Pre-reaction complex (case1)

C	0.1072800000	-0.8677090000	1.4300240000
H	1.0927890000	-0.9059420000	1.8993290000
N	-0.3743360000	0.5156370000	1.4968720000
C	-0.5755300000	1.2742040000	0.4230450000
C	-0.7277050000	1.2202240000	2.6258140000
H	-0.3449200000	0.9403600000	-0.6009360000
C	-1.1571130000	2.4389150000	2.2058480000
H	-0.6495580000	0.7999120000	3.6142750000
H	-1.5291210000	3.2892150000	2.7525200000
N	-1.0541820000	2.4468090000	0.8325910000
C	-1.4311890000	3.5499160000	-0.0417450000
H	-0.8835850000	4.4461170000	0.2488060000
H	-1.1805190000	3.2787390000	-1.0648720000
H	-2.5034560000	3.7250150000	0.0391580000
F	0.2216940000	-0.0624600000	-1.8682050000
P	-3.8321180000	-0.1272120000	-0.3708080000
F	-5.2054890000	0.7115350000	-0.3728320000
F	-4.5410830000	-1.3555510000	0.3911260000
F	-4.2248050000	-0.7865430000	-1.8188880000
F	-3.0836170000	1.0852070000	-1.1826290000
F	-2.4284450000	-0.9670130000	-0.4282640000
F	-3.3948680000	0.5311030000	1.0321800000
H	0.2204120000	-1.0801300000	0.3641490000

C	-0.8527540000	-1.8373050000	2.1062420000
H	-0.8532230000	-1.6619660000	3.1873260000
H	-1.8661510000	-1.6531100000	1.7423810000
C	-0.4527730000	-3.2822250000	1.8180820000
H	0.5749430000	-3.4520970000	2.1581700000
H	-0.4559700000	-3.4392910000	0.7339760000
C	-1.3916510000	-4.2800160000	2.4885530000
H	-2.4190260000	-4.1354250000	2.1427630000
H	-1.1034010000	-5.3099470000	2.2668440000
H	-1.3832420000	-4.1546120000	3.5749050000
K	-1.7758260000	-0.5645260000	-2.9712730000
C	2.2408940000	2.8291620000	-0.2271840000
C	2.1426140000	2.9408060000	1.1525840000
C	2.6337650000	1.9194470000	1.9648750000
C	3.2313030000	0.8056140000	1.3956400000
C	3.3378610000	0.6977420000	0.0048100000
C	2.8328780000	1.7081030000	-0.8061840000
H	1.8556780000	3.6135080000	-0.8685380000
H	1.6812590000	3.8149990000	1.5983280000
H	2.5568910000	1.9935990000	3.0432270000
H	3.6222890000	0.0209880000	2.0344350000
H	2.8869270000	1.6374860000	-1.8828510000
C	4.0389190000	-0.5247920000	-0.5550570000
Cl	3.2054470000	-2.0271170000	-0.0298120000
Cl	4.1239150000	-0.5453300000	-2.3328240000
Cl	5.7266260000	-0.5939860000	0.0710010000

Electronic Energy (Hartree)	Zero point correction (kcal/mol)	Electronic and Zero point correction (Hartree)	Gibbs free Energy (kcal/mol)	Electronic and Gibbs Free Energy (Hartree)	Number of Imaginary frequencies
-3714.090304	220.2401468	-3713.7393	144.657255	-3713.859778	0

Figure 1S. Transition state (case1)

C	-0.2637410000	-0.5171450000	2.0304690000
H	0.6081560000	-0.5357870000	2.6894210000
N	-0.5086750000	0.8755780000	1.6371380000
C	-0.3709600000	1.3228140000	0.3924210000
C	-0.9396170000	1.9052640000	2.4419820000
H	-0.0176690000	0.6984850000	-0.4293990000
C	-1.0638790000	2.9990200000	1.6444380000
H	-1.1205900000	1.7736750000	3.4954710000
H	-1.3778100000	4.0060990000	1.8620450000
N	-0.7048820000	2.6099140000	0.3733880000
C	-0.7535830000	3.4503430000	-0.8169880000
H	-0.1987590000	4.3691100000	-0.6303700000
H	-0.3014170000	2.9059930000	-1.6430500000
H	-1.7925680000	3.6808540000	-1.0510800000
F	0.7336100000	-0.8369060000	-1.0624090000
P	-3.4742870000	0.0184630000	-0.8388790000
F	-4.6248970000	0.9292130000	-1.4963400000
F	-4.5553540000	-0.7751720000	0.0496030000
F	-3.6428390000	-1.0792500000	-2.0444560000
F	-2.3429880000	0.7746070000	-1.7556130000
F	-2.2765440000	-0.9173930000	-0.2286510000

F	-3.2498920000	1.0943870000	0.3389810000
H	-0.0160620000	-1.0366520000	1.1015000000
C	-1.4781930000	-1.1302410000	2.7168310000
H	-1.6052350000	-0.6890220000	3.7108970000
H	-2.3729700000	-0.8965470000	2.1349380000
C	-1.3255440000	-2.6443020000	2.8390830000
H	-0.4036310000	-2.8775360000	3.3831930000
H	-1.2158360000	-3.0689360000	1.8353540000
C	-2.5188170000	-3.2834960000	3.5419330000
H	-3.4442420000	-3.0733440000	2.9983170000
H	-2.4068890000	-4.3677920000	3.6103110000
H	-2.6302050000	-2.8920170000	4.5568750000
K	-1.0106370000	-1.5434740000	-2.4844310000
C	2.5972040000	2.8548790000	-0.5473460000
C	2.5103550000	3.1144190000	0.8208070000
C	2.6140820000	2.0737730000	1.7427100000
C	2.7994460000	0.7745490000	1.3028500000
C	2.8801680000	0.5084370000	-0.0734740000
C	2.7818240000	1.5605210000	-0.9988840000
H	2.5186660000	3.6650810000	-1.2618060000
H	2.3672660000	4.1311380000	1.1690550000
H	2.5600840000	2.2781890000	2.8049730000
H	2.9148350000	-0.0248010000	2.0233830000
H	2.8346300000	1.3640460000	-2.0614010000
C	3.0440990000	-0.8471210000	-0.5376210000
Cl	2.7892900000	-2.1727400000	0.4764440000

Cl 3.3337840000 -1.2153050000 -2.1596090000

Cl 5.7277860000 -0.7481470000 0.0484470000

Electronic Energy (Hartree)	Zero point correction (kcal/mol)	Electronic and Zero point correction (Hartree)	Gibbs free Energy (kcal/mol)	Electronic and Gibbs Free Energy (Hartree)	Number of Imaginary frequencies
-3714.056354	219.7168038	-3713.7062	144.4068787	-3713.826227	1

Figure 1S. Post-reaction complex (case1)

C 1.3431070000 0.4343180000 1.2540480000

H 1.7159420000 -0.5698290000 1.4649510000

N 2.3541450000 1.0876280000 0.4130960000

C 2.0920860000 1.9623650000 -0.5517330000

C 3.7142810000 0.8940700000 0.4849940000

H 1.1073570000 2.2878660000 -0.8505140000

C 4.2731500000 1.6784490000 -0.4753720000

H 4.1373190000 0.1939200000 1.1903660000

H 5.2993400000 1.8245560000 -0.7679930000

N 3.2409340000 2.3347180000 -1.1091490000

C 3.3815320000 3.2560940000 -2.2330580000

H 3.8683190000 2.7397570000 -3.0592390000

H 2.3897690000 3.5853130000 -2.5346770000

H 3.9783160000 4.1125690000 -1.9237290000

F -2.0339150000 -2.0540640000 -1.1372710000

P -2.1630240000 1.9308320000 -0.3438160000

F -2.7773560000 3.3779010000 -0.6508210000

F -1.0646950000 2.5531910000 0.6531750000

F -3.2040480000 1.7344140000 0.9090330000

F	-3.2815160000	1.2262200000	-1.3122870000
F	-1.5985430000	0.4203990000	-0.0221000000
F	-1.1432180000	2.0496650000	-1.5851250000
H	0.4377840000	0.3639050000	0.6536930000
C	1.0820830000	1.2026940000	2.5411040000
H	1.9896480000	1.2093810000	3.1544680000
H	0.8312490000	2.2402010000	2.2994230000
C	-0.0700720000	0.5636370000	3.3131710000
H	0.1868750000	-0.4752420000	3.5487890000
H	-0.9538560000	0.5353650000	2.6653320000
C	-0.3936470000	1.3222650000	4.5960730000
H	-0.6798070000	2.3541810000	4.3737860000
H	-1.2180440000	0.8538560000	5.1384560000
H	0.4736280000	1.3495920000	5.2614020000
K	-3.8594570000	-0.8087580000	0.4159030000
C	1.1448420000	-0.1811300000	-2.8757160000
C	2.4478900000	-0.5435820000	-2.5539000000
C	2.6825490000	-1.5350590000	-1.6017770000
C	1.6161960000	-2.1753980000	-0.9872290000
C	0.3117680000	-1.8175640000	-1.3296390000
C	0.0666560000	-0.8166470000	-2.2644220000
H	0.9582710000	0.5993340000	-3.6045090000
H	3.2840870000	-0.0490560000	-3.0361180000
H	3.6960120000	-1.8095890000	-1.3341560000
H	1.8020600000	-2.9422240000	-0.2422840000
H	-0.9460540000	-0.5261250000	-2.5102590000

C -0.8399210000 -2.5431490000 -0.6980490000

Cl -0.8595710000 -2.3693330000 1.0886110000

Cl -0.8350250000 -4.2819070000 -1.1010910000

Cl 3.6396490000 -2.1374620000 2.4147090000

Electronic Energy (Hartree)	Zero point correction (kcal/mol)	Electronic and Zero point correction (Hartree)	Gibbs free Energy (kcal/mol)	Electronic and Gibbs Free Energy (Hartree)	Number of Imaginary frequencies
-3714.122471	221.0527716	-3713.7702	144.8630781	-3713.891617	0

Figure 1S. Pre-reaction complex (case2)

F -1.1893390000 0.1652000000 1.6742010000

P 3.1827150000 -1.3785190000 0.6328980000

F 2.4265720000 -1.3372360000 2.0843260000

F 2.2890690000 -2.6981420000 0.2557060000

F 1.9806210000 -0.4467260000 0.0294330000

F 4.0179060000 -0.0568920000 1.0229500000

F 3.8821000000 -1.4332310000 -0.8181800000

F 4.3298220000 -2.3235780000 1.2515620000

K -0.0942760000 -1.8631270000 1.2709890000

C -0.2989270000 -1.3805870000 -1.8795150000

C -0.3615540000 -2.7675800000 -1.7976150000

C -1.4753770000 -3.3728110000 -1.2171020000

C -2.5163450000 -2.5937990000 -0.7251670000

C -2.4508200000 -1.1993190000 -0.8059660000

C -1.3394620000 -0.5947600000 -1.3874470000

H 0.5664110000 -0.9014060000 -2.3213060000

H 0.4515830000 -3.3731160000 -2.1791470000

H	-1.5381900000	-4.4520950000	-1.1481410000
H	-3.3811890000	-3.0770940000	-0.2854270000
H	-1.2684610000	0.4821650000	-1.4585450000
C	-3.6475480000	-0.4019630000	-0.3226680000
Cl	-4.1704600000	-0.9275850000	1.3021840000
Cl	-3.3786250000	1.3547490000	-0.2926920000
Cl	-5.0225510000	-0.7101320000	-1.4591080000
C	1.3254160000	2.3082290000	-1.4030630000
H	1.1345590000	2.7252480000	-2.3936150000
N	0.4661010000	3.0231650000	-0.4528570000
C	-0.2647130000	2.4464970000	0.4993090000
C	0.3113540000	4.3913040000	-0.3733040000
H	-0.4065100000	1.3807970000	0.7327380000
C	-0.5447330000	4.6322600000	0.6536900000
H	0.8134050000	5.0606040000	-1.0518670000
H	-0.9372370000	5.5530960000	1.0509760000
N	-0.8892300000	3.4066130000	1.1783530000
C	-1.7915710000	3.1495370000	2.3006310000
H	-2.7631620000	3.5947140000	2.0893800000
H	-1.8707440000	2.0654660000	2.3928260000
H	-1.3710240000	3.5863260000	3.2056780000
H	1.0047440000	1.2659330000	-1.3925410000
C	2.7978720000	2.4124240000	-1.0321200000
H	3.0998630000	3.4651970000	-1.0192190000
H	2.9382230000	2.0110880000	-0.0242880000
C	3.6580790000	1.6297030000	-2.0208490000

H	3.5104570000	2.0327610000	-3.0286840000
H	3.3174820000	0.5905150000	-2.0378580000
C	5.1392340000	1.6729250000	-1.6582810000
H	5.3034130000	1.2606410000	-0.6600880000
H	5.7357620000	1.0912000000	-2.3647710000
H	5.5146510000	2.7002450000	-1.6665470000

Electronic Energy (Hartree)	Zero point correction (kcal/mol)	Electronic and Zero point correction (Hartree)	Gibbs free Energy (kcal/mol)	Electronic and Gibbs Free Energy (Hartree)	Number of Imaginary frequencies
-3714.094312	220.6273201	-3713.7427	146.7393315	-3713.860468	0

Figure 1S. Transition state (case2)

F	1.4641990000	0.3870280000	-1.2587230000
P	-2.8538950000	-1.7887300000	-0.3630650000
F	-2.4079930000	-1.6595030000	-1.9359390000
F	-1.6751050000	-2.9105740000	-0.1761490000
F	-1.7426360000	-0.6323590000	-0.0111290000
F	-3.9750370000	-0.6566370000	-0.5816970000
F	-3.2245420000	-1.9107040000	1.1985660000
F	-3.9025800000	-2.9467600000	-0.7427910000
K	0.2588280000	-1.6884970000	-1.5684240000
C	0.7965080000	-2.2358090000	1.6772850000
C	1.2064980000	-3.4559330000	1.1412230000
C	2.3048290000	-3.5074070000	0.2834530000
C	2.9851410000	-2.3445540000	-0.0519060000
C	2.5652580000	-1.1190160000	0.4796800000
C	1.4730690000	-1.0691410000	1.3553580000
H	-0.0614460000	-2.1915540000	2.3365850000
H	0.6694570000	-4.3632750000	1.3900640000
H	2.6352630000	-4.4548840000	-0.1238100000
H	3.8463600000	-2.3953380000	-0.7055460000

H	1.1361400000	-0.1250980000	1.7625990000
C	3.2032600000	0.1230880000	0.0462980000
Cl	4.2431740000	0.1808940000	-1.2911250000
Cl	2.8871600000	1.6132240000	0.7982160000
Cl	5.2080560000	-0.4066360000	1.7382460000
C	-1.9898930000	2.3562200000	1.2772800000
H	-1.9975010000	2.8374640000	2.2565230000
N	-1.0703380000	3.1224960000	0.4253420000
C	-0.1343590000	2.6039600000	-0.3653940000
C	-1.0573150000	4.4936740000	0.2918950000
H	0.1339120000	1.5590780000	-0.4883820000
C	-0.0776780000	4.7951390000	-0.5996530000
H	-1.7349120000	5.1240150000	0.8431730000
H	0.2702210000	5.7397020000	-0.9825430000
N	0.4833420000	3.6018300000	-0.9939600000
C	1.5829070000	3.4361730000	-1.9453050000
H	2.4505650000	3.9874250000	-1.5847040000
H	1.8027640000	2.3709100000	-2.0042070000
H	1.2738560000	3.8201100000	-2.9164510000
H	-1.5690720000	1.3562060000	1.3811560000
C	-3.3891640000	2.2937530000	0.6815990000
H	-3.7680310000	3.3113880000	0.5379810000
H	-3.3384100000	1.8143630000	-0.3007850000
C	-4.3341220000	1.5085230000	1.5871840000
H	-4.3455600000	1.9652760000	2.5827350000
H	-3.9495930000	0.4913620000	1.7041860000
C	-5.7508940000	1.4598010000	1.0245460000
H	-5.7547400000	1.0029690000	0.0322490000
H	-6.4119080000	0.8738460000	1.6671360000
H	-6.1713420000	2.4657120000	0.9371540000

Electronic	Zero point	Electronic and	Gibbs free	Electronic and	Number of

Energy (Hartree)	correction (kcal/mol)	Zero point correction (Hartree)	Energy (kcal/mol)	Gibbs Free Energy (Hartree)	Imaginary frequencies
-3714.056365	219.5674566	-3713.7065	142.9905898	-3713.828495	1

Figure 1S. Post-reaction complex (case2)

F	1.2518050000	0.6095720000	-1.3220230000
P	-2.1194620000	-2.0515380000	-0.3353220000
F	-1.6539160000	-2.1964740000	-1.9015420000
F	-1.0717550000	-3.2573770000	0.0305690000
F	-0.8735510000	-0.9912840000	-0.1398180000
F	-3.0882670000	-0.8248880000	-0.7293900000
F	-2.4978720000	-1.8962270000	1.2182960000
F	-3.2949820000	-3.1135380000	-0.5677280000
K	0.9836230000	-2.4374440000	-1.4182040000
C	2.0154160000	-2.2746550000	1.6664910000
C	3.0400530000	-3.0518670000	1.1306180000
C	3.8673800000	-2.5219270000	0.1435430000
C	3.6672720000	-1.2235040000	-0.3181920000
C	2.6366230000	-0.4492130000	0.2153870000
C	1.8117270000	-0.9762710000	1.2115550000
H	1.3695900000	-2.6758670000	2.4378400000
H	3.1981240000	-4.0625330000	1.4872520000
H	4.6758670000	-3.1137280000	-0.2676440000
H	4.3223450000	-0.8260720000	-1.0820550000
H	1.0042060000	-0.3885930000	1.6276800000
C	2.2563470000	0.8706500000	-0.4162890000
Cl	3.5586100000	1.6476880000	-1.3360980000
Cl	1.5904900000	2.0451320000	0.7424730000
Cl	5.3685240000	0.9208090000	2.3184870000
C	-2.6032290000	1.5914230000	1.2642990000
H	-2.4001820000	1.9763530000	2.2641520000
N	-1.8301760000	2.4201520000	0.3284380000

C	-1.3732970000	2.0202110000	-0.8549270000
C	-1.5135960000	3.7480740000	0.5000280000
H	-1.4632580000	1.0230740000	-1.2552850000
C	-0.8436890000	4.1427650000	-0.6144700000
H	-1.7881850000	4.2842080000	1.3931700000
H	-0.4086690000	5.0899270000	-0.8860570000
N	-0.7699290000	3.0484050000	-1.4446540000
C	-0.0922780000	3.0160690000	-2.7383430000
H	0.9741830000	3.1802390000	-2.5874250000
H	-0.2589350000	2.0426410000	-3.1928340000
H	-0.5054740000	3.7980580000	-3.3728420000
H	-2.2110310000	0.5772450000	1.1988130000
C	-4.0909490000	1.6281580000	0.9429760000
H	-4.4552830000	2.6571400000	1.0311750000
H	-4.2366110000	1.3109990000	-0.0939820000
C	-4.8768150000	0.7090620000	1.8743930000
H	-4.7186040000	1.0221520000	2.9117990000
H	-4.4822290000	-0.3079560000	1.7858830000
C	-6.3681630000	0.7151460000	1.5537120000
H	-6.5456520000	0.3753330000	0.5296250000
H	-6.9209910000	0.0560220000	2.2264740000
H	-6.7849800000	1.7214830000	1.6508730000

Electronic Energy (Hartree)	Zero point correction (kcal/mol)	Electronic and Zero point correction (Hartree)	Gibbs free Energy (kcal/mol)	Electronic and Gibbs Free Energy (Hartree)	Number of Imaginary frequencies
-3714.113262	221.2673798	-3713.7607	145.4303467	-3713.881504	0

Figure 2S. Pre-reaction complex (case1)

F	0.7373390000	-0.3650630000	1.8878410000
K	0.3898220000	1.9577470000	1.9447120000
C	0.0736660000	2.1271990000	-1.2610460000
C	0.7031970000	3.3279590000	-0.9503410000

C	2.0166700000	3.3150400000	-0.4839670000
C	2.6924480000	2.1096580000	-0.3311640000
C	2.0570960000	0.9034940000	-0.6403910000
C	0.7459820000	0.9162830000	-1.1103520000
H	-0.9488510000	2.1272850000	-1.6189400000
H	0.1747740000	4.2664910000	-1.0687350000
H	2.5209270000	4.2429500000	-0.2416000000
H	3.7177620000	2.1160420000	0.0196960000
H	0.2374170000	-0.0056980000	-1.3595850000
C	2.8681330000	-0.3751720000	-0.5527860000
Cl	3.7998600000	-0.4659540000	0.9654700000
Cl	1.8943360000	-1.8543820000	-0.7090390000
Cl	4.0573120000	-0.3728630000	-1.9192470000
C	-2.7302350000	-0.5109050000	-1.0432530000
H	-2.5732820000	-0.5989550000	-2.1209770000
N	-2.1681110000	-1.7107380000	-0.4105840000
C	-1.2121190000	-1.7104720000	0.5163790000
C	-2.5461580000	-3.0129240000	-0.6662800000
H	-0.6442400000	-0.8723850000	0.9420710000
C	-1.7884520000	-3.8078910000	0.1325880000
H	-3.3075700000	-3.2546970000	-1.3887810000
H	-1.7597440000	-4.8784460000	0.2483990000
N	-0.9671750000	-2.9741470000	0.8583020000
C	0.0413160000	-3.3660120000	1.8425460000
H	0.7202680000	-4.0886350000	1.3911030000
H	0.5717360000	-2.4539770000	2.1215420000

H -0.4513390000 -3.8104340000 2.7069060000

H -2.1611730000 0.3373670000 -0.6606840000

C -4.2029290000 -0.3284980000 -0.7089880000

H -4.7708330000 -1.2122450000 -1.0195920000

H -4.3021820000 -0.2281320000 0.3762570000

C -4.7616980000 0.9155300000 -1.3945340000

H -4.6924040000 0.7937260000 -2.4810210000

H -4.1388470000 1.7741830000 -1.1238540000

C -6.2078740000 1.1870690000 -0.9932750000

H -6.2824230000 1.3588700000 0.0842440000

H -6.6028040000 2.0696300000 -1.5016430000

H -6.8513750000 0.3386620000 -1.2437470000

Cl -2.5189050000 2.1472480000 1.4706980000

Electronic Energy (Hartree)	Zero point correction (kcal/mol)	Electronic and Zero point correction (Hartree)	Gibbs free Energy (kcal/mol)	Electronic and Gibbs Free Energy (Hartree)	Number of Imaginary frequencies
-3233.713955	208.2264774	-3233.3821	143.0332604	-3233.486016	0

Figure 2S. Transition state (case1)

F 0.8250850000 -0.3261890000 1.2010040000

K 0.6588630000 2.0188290000 1.8254860000

C 1.6182450000 2.7512470000 -1.2389170000

C 2.5777280000 3.4599580000 -0.5187660000

C 3.4898200000 2.7729160000 0.2819210000

C 3.4414970000 1.3876070000 0.3700360000

C 2.4716960000 0.6815060000 -0.3482240000

C 1.5604820000 1.3656500000 -1.1566790000

H	0.9017300000	3.2766800000	-1.8584130000
H	2.6175450000	4.5406650000	-0.5837030000
H	4.2457890000	3.3153230000	0.8367220000
H	4.1635970000	0.8640320000	0.9832520000
H	0.7972970000	0.8296270000	-1.7058150000
C	2.3487240000	-0.7792480000	-0.1786050000
Cl	3.2009260000	-1.6286880000	1.0354230000
Cl	1.2508600000	-1.7138640000	-1.1010320000
Cl	4.2184030000	-1.0709030000	-1.8905370000
C	-3.4092460000	-0.3694670000	-1.1278810000
H	-3.6510970000	-0.7244840000	-2.1313930000
N	-2.8324180000	-1.5050470000	-0.3954610000
C	-1.7349140000	-1.4684190000	0.3573060000
C	-3.3660630000	-2.7741370000	-0.3459530000
H	-1.0734490000	-0.6239680000	0.5150390000
C	-2.5551040000	-3.5134910000	0.4531570000
H	-4.2607960000	-3.0348610000	-0.8865170000
H	-2.6001350000	-4.5474660000	0.7516950000
N	-1.5460540000	-2.6797560000	0.8791660000
C	-0.4311000000	-3.0636510000	1.7451010000
H	0.1152810000	-3.8814120000	1.2757830000
H	0.2117800000	-2.1922830000	1.8577250000
H	-0.8236190000	-3.3846620000	2.7091540000
H	-2.6332600000	0.3935370000	-1.1930510000
C	-4.6366330000	0.1862260000	-0.4216110000
H	-5.3899780000	-0.6017750000	-0.3132990000

H	-4.3408890000	0.5126510000	0.5798760000
C	-5.2210630000	1.3664470000	-1.1926550000
H	-5.5374690000	1.0320270000	-2.1866380000
H	-4.4348080000	2.1139230000	-1.3394420000
C	-6.3984800000	1.9992130000	-0.4580750000
H	-6.0847700000	2.3766050000	0.5193850000
H	-6.8161030000	2.8360870000	-1.0224140000
H	-7.1980680000	1.2705480000	-0.2961780000
Cl	-1.9664800000	2.3832570000	0.6201520000

Electronic Energy (Hartree)	Zero point correction (kcal/mol)	Electronic and Zero point correction (Hartree)	Gibbs free Energy (kcal/mol)	Electronic and Gibbs Free Energy (Hartree)	Number of Imaginary frequencies
-3233.677149	207.3335314	-3233.3467	140.3142617	-3233.453544	1

Figure 2S. Post-reaction complex (case1)

F	0.7922840000	0.7748200000	-1.5350630000
K	1.0176140000	-2.6054870000	-2.0382190000
C	0.9731690000	-2.3861950000	1.1343750000
C	2.1697570000	-3.0884570000	0.9852480000
C	3.2581370000	-2.4822450000	0.3656160000
C	3.1614670000	-1.1746890000	-0.1093370000
C	1.9649150000	-0.4777970000	0.0383680000
C	0.8690110000	-1.0855080000	0.6600500000
H	0.1201820000	-2.8507400000	1.6143500000
H	2.2529920000	-4.1026090000	1.3573490000
H	4.1930190000	-3.0180650000	0.2570090000
H	4.0223650000	-0.7148220000	-0.5756140000

H	-0.0645910000	-0.5460440000	0.7694980000
C	1.7339390000	0.8934710000	-0.5517920000
Cl	3.1616600000	1.6268840000	-1.3043170000
Cl	1.0649100000	2.0327050000	0.6523530000
Cl	4.5946150000	0.5304780000	3.0098370000
C	-2.9358750000	0.0040710000	1.1714970000
H	-2.6499350000	0.0784120000	2.2219140000
N	-2.4600920000	1.2280870000	0.5108100000
C	-1.9933390000	1.2950120000	-0.7340280000
C	-2.4976950000	2.4980160000	1.0409920000
H	-1.8552960000	0.4449750000	-1.3901080000
C	-2.0330800000	3.3404870000	0.0818920000
H	-2.8429770000	2.6837410000	2.0445770000
H	-1.8830090000	4.4072580000	0.0831940000
N	-1.7302410000	2.5683750000	-1.0166660000
C	-1.1214970000	3.0589910000	-2.2507140000
H	-0.0992580000	3.3782190000	-2.0466040000
H	-1.1213210000	2.2540120000	-2.9821200000
H	-1.7073600000	3.8974820000	-2.6231140000
H	-2.4019590000	-0.8314300000	0.7152840000
C	-4.4396720000	-0.1695200000	1.0114470000
H	-4.9546230000	0.6955690000	1.4429160000
H	-4.6799710000	-0.1987670000	-0.0569100000
C	-4.9165450000	-1.4536580000	1.6846490000
H	-4.6477180000	-1.4272250000	2.7459370000
H	-4.3869490000	-2.3054700000	1.2446780000

C -6.4218770000 -1.6506260000 1.5382310000

H -6.7065940000 -1.7037020000 0.4838320000

H -6.7494190000 -2.5738040000 2.0213210000

H -6.9701220000 -0.8207360000 1.9927740000

Cl -1.7991400000 -2.0441100000 -1.7482490000

Electronic Energy (Hartree)	Zero point correction (kcal/mol)	Electronic and Zero point correction (Hartree)	Gibbs free Energy (kcal/mol)	Electronic and Gibbs Free Energy (Hartree)	Number of Imaginary frequencies
-3233.737959	208.6764017	-3233.4054	138.9877067	-3233.516468	0

Figure 2S. Pre-reaction complex (case2)

C 0.6271020000 -0.5956840000 -1.1797060000

H -0.1756240000 -0.5014160000 -1.9127020000

N 1.2635480000 0.7181870000 -1.0431080000

C 1.6058920000 1.2542040000 0.1258610000

C 1.6700650000 1.5540240000 -2.0596440000

H 1.3874390000 0.7673110000 1.0800010000

C 2.2619770000 2.6258510000 -1.4712830000

H 1.4986900000 1.3186190000 -3.0970240000

H 2.7117430000 3.5095520000 -1.8922670000

N 2.2062830000 2.4160810000 -0.1106500000

C 2.7397100000 3.3077300000 0.9101410000

H 2.5654270000 2.8575830000 1.8852180000

H 3.8100470000 3.4360810000 0.7529460000

H 2.2351330000 4.2721430000 0.8513560000

F 0.7753090000 -0.6943580000 1.9108880000

H 0.2065810000 -0.8221040000 -0.1969330000

C	1.6343340000	-1.6608070000	-1.5956940000
H	1.9824840000	-1.4658760000	-2.6157690000
H	2.5207960000	-1.5834850000	-0.9547480000
C	1.0279690000	-3.0593190000	-1.5081130000
H	0.1570710000	-3.1171580000	-2.1700790000
H	0.6525120000	-3.2280920000	-0.4916440000
C	2.0314080000	-4.1464120000	-1.8801100000
H	2.9023900000	-4.1200000000	-1.2180790000
H	1.5851590000	-5.1406660000	-1.8077490000
H	2.3899520000	-4.0117350000	-2.9042450000
K	2.8072980000	-1.8802660000	1.8641390000
C	-1.0796000000	2.6556450000	0.9214070000
C	-0.9912290000	3.1842190000	-0.3583390000
C	-1.5990360000	2.5194900000	-1.4227880000
C	-2.3026130000	1.3464560000	-1.2002910000
C	-2.3970580000	0.8180330000	0.0920520000
C	-1.7773240000	1.4707840000	1.1521080000
H	-0.6011370000	3.1573050000	1.7550120000
H	-0.4468380000	4.1056210000	-0.5328620000
H	-1.5302720000	2.9191360000	-2.4277370000
H	-2.7862120000	0.8462880000	-2.0322860000
H	-1.8248190000	1.0756510000	2.1568010000
C	-3.2396590000	-0.4272630000	0.2863960000
Cl	-2.7102400000	-1.7377460000	-0.8222380000
Cl	-3.1866700000	-1.0718490000	1.9442320000
Cl	-4.9585060000	-0.0501470000	-0.1003450000

Cl	4.6720390000	-0.0415560000	0.5289680000

Electronic Energy (Hartree)	Zero point correction (kcal/mol)	Electronic and Zero point correction (Hartree)	Gibbs free Energy (kcal/mol)	Electronic and Gibbs Free Energy (Hartree)	Number of Imaginary frequencies
-3233.713525	208.0219093	-3233.382	141.0534679	-3233.488741	0

Figure 2S. Transition state (case2)

C	1.2666250000	-0.4705320000	-1.5935330000
H	0.7765440000	-0.4559780000	-2.5693680000
N	1.4811140000	0.9177060000	-1.1739480000
C	1.1787680000	1.3778700000	0.0378810000
C	2.0500480000	1.9313450000	-1.9124440000
H	0.7002120000	0.7680510000	0.8029110000
C	2.0769980000	3.0295040000	-1.1125840000
H	2.3739010000	1.7862490000	-2.9297030000
H	2.4338260000	4.0303280000	-1.2902800000
N	1.5288080000	2.6582510000	0.0958110000
C	1.3931470000	3.5132410000	1.2682870000
H	0.8549320000	2.9620910000	2.0370850000
H	2.3839100000	3.7805570000	1.6347010000
H	0.8389610000	4.4116950000	0.9978930000
F	-0.0314330000	-0.8972610000	1.1987920000
H	0.5898070000	-0.8965740000	-0.8488770000
C	2.5742640000	-1.2520600000	-1.6413530000
H	3.2212830000	-0.8496310000	-2.4275790000
H	3.1128630000	-1.1060160000	-0.6969110000
C	2.3164420000	-2.7368260000	-1.8895280000
H	1.8046390000	-2.8583350000	-2.8499580000
H	1.6275160000	-3.1218280000	-1.1273110000
C	3.6046270000	-3.5533100000	-1.8816440000
H	4.1181270000	-3.4634270000	-0.9197950000
H	3.4048440000	-4.6121250000	-2.0596730000

H	4.2903680000	-3.2045120000	-2.6584680000
K	2.0122140000	-1.8390950000	1.9547150000
C	-1.8679350000	2.8466190000	0.4428110000
C	-1.5759980000	3.0963050000	-0.8989630000
C	-1.5474470000	2.0510500000	-1.8218150000
C	-1.8042890000	0.7561720000	-1.4090770000
C	-2.0898920000	0.4975740000	-0.0563850000
C	-2.1248100000	1.5569000000	0.8684600000
H	-1.8935840000	3.6612880000	1.1559860000
H	-1.3763020000	4.1102780000	-1.2271670000
H	-1.3336590000	2.2497140000	-2.8648510000
H	-1.8161710000	-0.0457290000	-2.1360400000
H	-2.3375690000	1.3693030000	1.9124340000
C	-2.3344370000	-0.8476160000	0.3839230000
Cl	-1.9875840000	-2.1818840000	-0.5881990000
Cl	-2.8214360000	-1.2052350000	1.9548830000
Cl	-4.9619520000	-0.7009780000	-0.5434640000
Cl	3.8009880000	0.4818410000	1.8226050000

Electronic Energy (Hartree)	Zero point correction (kcal/mol)	Electronic and Zero point correction (Hartree)	Gibbs free Energy (kcal/mol)	Electronic and Gibbs Free Energy (Hartree)	Number of Imaginary frequencies
-3233.677959	207.2274823	-3233.3477	139.0937558	-3233.4563	1

Figure 2S. Post-reaction complex (case2)

C	1.1680330000	1.3513450000	0.0855830000
H	1.0709770000	1.1206900000	1.1484380000
N	2.1646570000	0.4217260000	-0.4595390000
C	2.0173180000	-0.2892730000	-1.5724920000
C	3.3916260000	0.1369210000	0.0957070000
H	1.1436800000	-0.2698990000	-2.2104480000
C	3.9889150000	-0.7721100000	-0.7192820000
H	3.7000080000	0.5794720000	1.0296740000
H	4.9431230000	-1.2677370000	-0.6543760000

N	3.1150950000	-1.0188780000	-1.7550440000
C	3.3208340000	-1.9868260000	-2.8273240000
H	2.4616420000	-1.9524150000	-3.4937980000
H	4.2249970000	-1.7292390000	-3.3768930000
H	3.4169410000	-2.9835200000	-2.3972720000
F	-3.0089070000	-1.0589890000	-0.3466260000
H	0.2324390000	1.1335550000	-0.4305710000
C	1.5713980000	2.8035650000	-0.1183300000
H	2.5249720000	2.9935170000	0.3868520000
H	1.7172050000	2.9943300000	-1.1874270000
C	0.4967700000	3.7350090000	0.4371770000
H	0.3376870000	3.5059630000	1.4968190000
H	-0.4503110000	3.5333130000	-0.0768680000
C	0.8687760000	5.2052230000	0.2755860000
H	1.0129350000	5.4571860000	-0.7788500000
H	0.0895070000	5.8589730000	0.6739010000
H	1.7987240000	5.4309680000	0.8047140000
K	-3.7169130000	1.2238150000	-1.6609620000
C	0.4816930000	-3.1274620000	-0.7813280000
C	1.5057360000	-2.9900110000	0.1504950000
C	1.3299100000	-2.1749910000	1.2667280000
C	0.1274800000	-1.5092270000	1.4612570000
C	-0.8971950000	-1.6596590000	0.5279980000
C	-0.7274790000	-2.4636310000	-0.5972990000
H	0.6175210000	-3.7545680000	-1.6550160000
H	2.4455110000	-3.5116560000	0.0047840000
H	2.1269430000	-2.0427720000	1.9887560000
H	0.0068950000	-0.8729670000	2.3320870000
H	-1.5256300000	-2.5765150000	-1.3197680000
C	-2.2331690000	-1.0216100000	0.7724090000
Cl	-2.1095830000	0.7113430000	1.2238830000
Cl	-3.1497080000	-1.8765660000	2.0449550000

Cl 2.2751110000 0.7488300000 3.4729370000

Cl -1.1109020000 0.8509940000 -2.7915430000

Electronic Energy (Hartree)	Zero point correction (kcal/mol)	Electronic and Zero point correction (Hartree)	Gibbs free Energy (kcal/mol)	Electronic and Gibbs Free Energy (Hartree)	Number of Imaginary frequencies
-3233.745589	208.5904329	-3233.4132	139.1828621	-3233.523787	0

Figure 3S. Pre-reaction complex (case1)

C 0.1845370000 2.3874100000 1.2974030000

H 0.3580080000 1.5371580000 1.9605830000

N 1.4611690000 2.7210700000 0.6487880000

C 1.6377400000 2.7427940000 -0.6702980000

C 2.6417600000 3.0640380000 1.2694960000

H 0.8454550000 2.4552840000 -1.3716820000

C 3.5485850000 3.2948640000 0.2842970000

H 2.7347190000 3.1049900000 2.3417220000

H 4.5864950000 3.5794640000 0.3249900000

N 2.9007100000 3.0860080000 -0.9132800000

C 3.4797120000 3.2716520000 -2.2409570000

H 4.5136650000 2.9371670000 -2.2198710000

H 2.9160200000 2.6701020000 -2.9506580000

H 3.4288600000 4.3242760000 -2.5174520000

F -0.6892140000 1.6570130000 -1.6285550000

P 3.4010060000 -0.5956080000 -1.2139600000

F 4.4689070000 0.5563340000 -1.5306220000

F 4.4997520000 -1.4050690000 -0.3147770000

F 2.9379150000 0.1902350000 0.1465340000

F 2.2497500000 0.1843000000 -2.0461650000

F	2.2984480000	-1.7585590000	-0.8280170000
F	3.8288850000	-1.4211050000	-2.5178180000
H	-0.4808140000	2.0830930000	0.4841960000
C	-0.3792660000	3.5764700000	2.0644160000
H	0.3108000000	3.8590760000	2.8667860000
H	-0.4604470000	4.4321040000	1.3858890000
C	-1.7499920000	3.2524110000	2.6538160000
H	-1.6794790000	2.3423540000	3.2606090000
H	-2.4444790000	3.0307750000	1.8378590000
C	-2.2967020000	4.3975360000	3.5002510000
H	-2.3875890000	5.3109490000	2.9058200000
H	-3.2843450000	4.1580390000	3.9003490000
H	-1.6345460000	4.6112020000	4.3439000000
K	-0.2123110000	-0.6211680000	-1.1457410000
C	-2.1369280000	-0.8329510000	-3.8529760000
C	-1.9557750000	-2.1801960000	-3.5653430000
C	-2.4573860000	-2.7078110000	-2.3740660000
C	-3.1289900000	-1.8865040000	-1.4804550000
C	-3.2990110000	-0.5262380000	-1.7670140000
C	-2.8051020000	-0.0001870000	-2.9555390000
H	-1.7490330000	-0.4145380000	-4.7737590000
H	-1.4274740000	-2.8198040000	-4.2624470000
H	-2.3216310000	-3.7563280000	-2.1381240000
H	-3.5008890000	-2.2993000000	-0.5496890000
H	-2.9050910000	1.0505640000	-3.1814230000
C	-3.9668510000	0.3315740000	-0.7099510000

Cl -2.9878260000 0.2737770000 0.7938180000

Cl -4.1710190000 2.0318100000 -1.1821070000

Cl -5.5973230000 -0.3275260000 -0.3269490000

P -0.2393280000 -2.2027710000 2.1420160000

F -1.2359840000 -1.1341220000 2.8036560000

F -0.9754150000 -3.4189990000 2.8734570000

F -1.1876400000 -2.3451790000 0.8392170000

F 0.5620010000 -0.9708280000 1.3802930000

F 0.8315660000 -3.2347710000 1.4567310000

F 0.8023660000 -2.0306320000 3.3866990000

K 3.0574850000 -1.8748460000 1.8582600000

Electronic Energy (Hartree)	Zero point correction (kcal/mol)	Electronic and Zero point correction (Hartree)	Gibbs free Energy (kcal/mol)	Electronic and Gibbs Free Energy (Hartree)	Number of Imaginary frequencies
-5254.728099	233.4178463	-5254.3561	141.5473179	-5254.502529	0

Figure 3S. Transition state (case1)

C -0.2666320000 -2.3959320000 1.6525540000

H -0.7841400000 -1.8239370000 2.4253740000

N -1.2763050000 -2.8338020000 0.6796060000

C -1.1578400000 -2.7066490000 -0.6388700000

C -2.4766840000 -3.4439300000 0.9657740000

H -0.3192230000 -2.2069550000 -1.1167480000

C -3.0896430000 -3.6826050000 -0.2225790000

H -2.7863660000 -3.6445390000 1.9778030000

H -4.0395700000 -4.1330930000 -0.4565170000

N -2.2473230000 -3.2159030000 -1.2075460000

C	-2.5024110000	-3.2827360000	-2.6434780000
H	-3.4413420000	-2.7784470000	-2.8616540000
H	-1.6860910000	-2.7846740000	-3.1608050000
H	-2.5535350000	-4.3265880000	-2.9498490000
F	1.0158110000	-0.9447070000	-0.8354700000
P	-3.4929430000	0.2847010000	-1.1779640000
F	-4.3377470000	-1.0458900000	-1.4552950000
F	-4.6727670000	0.8828350000	-0.2245890000
F	-2.8233090000	-0.3908400000	0.1518540000
F	-2.2490230000	-0.2632350000	-2.0671160000
F	-2.6044310000	1.6406150000	-0.8420920000
F	-4.1219360000	1.0133240000	-2.4565920000
H	0.4073070000	-1.7342700000	1.1045770000
C	0.4777000000	-3.5834250000	2.2501550000
H	-0.2368320000	-4.2634000000	2.7267240000
H	0.9668340000	-4.1385520000	1.4423260000
C	1.5181640000	-3.1311150000	3.2713120000
H	1.0229070000	-2.5606740000	4.0642120000
H	2.2243910000	-2.4490250000	2.7875720000
C	2.2763420000	-4.3082780000	3.8766020000
H	2.8019970000	-4.8719490000	3.1008650000
H	3.0163470000	-3.9704010000	4.6051900000
H	1.5922190000	-4.9939190000	4.3843020000
K	-0.0191260000	1.1679980000	-1.3277470000
C	2.6343920000	0.7293580000	-4.0101120000
C	2.6379890000	2.1047160000	-3.7810160000

C	2.8890470000	2.6014540000	-2.5017470000
C	3.1209110000	1.7284080000	-1.4498020000
C	3.0986590000	0.3452750000	-1.6783210000
C	2.8665550000	-0.1528830000	-2.9665510000
H	2.4421400000	0.3433400000	-5.0034500000
H	2.4524610000	2.7897340000	-4.5997510000
H	2.9110200000	3.6695280000	-2.3237680000
H	3.3337350000	2.1177250000	-0.4628680000
H	2.8349270000	-1.2193020000	-3.1443980000
C	3.2099580000	-0.5756850000	-0.5549450000
Cl	2.9420620000	-0.0472880000	1.0280910000
Cl	3.4775300000	-2.2343830000	-0.7586860000
Cl	5.8709000000	-0.0634190000	-0.4281760000
P	0.0982060000	2.5563630000	1.9410570000
F	1.1831920000	1.7215280000	2.7718350000
F	0.6602750000	3.9453470000	2.4960690000
F	1.0819660000	2.6232120000	0.6515340000
F	-0.5331090000	1.1517690000	1.3426100000
F	-1.0534000000	3.3427970000	1.0787880000
F	-0.9640010000	2.4481160000	3.1751750000
K	-3.1473910000	1.7782230000	1.7969520000

Electronic Energy (Hartree)	Zero point correction (kcal/mol)	Electronic and Zero point correction (Hartree)	Gibbs free Energy (kcal/mol)	Electronic and Gibbs Free Energy (Hartree)	Number of Imaginary frequencies
-5254.688083	232.5387055	-5254.3175	138.6406939	-5254.467146	1

Figure 3S. Post-reaction complex (case1)

C	1.5336690000	2.6737610000	-0.7428060000

H	2.1594640000	2.5709440000	0.1458300000
N	2.0302750000	1.7223630000	-1.7460650000
C	1.3025880000	0.7798280000	-2.3347110000
C	3.3169290000	1.6523640000	-2.2302850000
H	0.2596680000	0.5871200000	-2.1439900000
C	3.3466660000	0.6349200000	-3.1299340000
H	4.0900710000	2.3190030000	-1.8869560000
H	4.1480860000	0.2373510000	-3.7295900000
N	2.0777790000	0.1046500000	-3.1771910000
C	1.6420190000	-0.9867970000	-4.0455850000
H	2.3443470000	-1.8114010000	-3.9505900000
H	0.6520760000	-1.3057630000	-3.7275400000
H	1.6075800000	-0.6342100000	-5.0753490000
F	-1.3944720000	0.8165840000	-0.5092170000
P	2.7304040000	-2.2978610000	-0.4431590000
F	3.4381150000	-2.2831120000	-1.8763100000
F	4.1446880000	-2.4920730000	0.3248780000
F	2.9269320000	-0.6842910000	-0.3054640000
F	1.2629210000	-2.0457190000	-1.1069160000
F	1.9894060000	-2.2739150000	1.0472530000
F	2.5067830000	-3.8817100000	-0.5116640000
H	0.5248570000	2.3562720000	-0.4837550000
C	1.5379830000	4.1035550000	-1.2628880000
H	2.5596130000	4.4014130000	-1.5211930000
H	0.9399610000	4.1520790000	-2.1788550000
C	0.9673400000	5.0596030000	-0.2173380000

H	1.5506830000	4.9758910000	0.7057870000
H	-0.0544980000	4.7529580000	0.0292290000
C	0.9700580000	6.5057090000	-0.7025600000
H	0.3729870000	6.6117610000	-1.6124240000
H	0.5542990000	7.1771160000	0.0516870000
H	1.9865610000	6.8401890000	-0.9275080000
K	-0.5823490000	-2.0141900000	0.9461600000
C	-2.8830360000	-3.0546700000	-1.0305600000
C	-3.4476240000	-3.4697270000	0.1758240000
C	-3.8422140000	-2.5244730000	1.1170280000
C	-3.6722060000	-1.1639980000	0.8650010000
C	-3.1055970000	-0.7509200000	-0.3380960000
C	-2.7089120000	-1.7001210000	-1.2874930000
H	-2.5804850000	-3.7833090000	-1.7726340000
H	-3.5864790000	-4.5255700000	0.3749000000
H	-4.2903050000	-2.8383980000	2.0516380000
H	-3.9831400000	-0.4444760000	1.6100140000
H	-2.2706020000	-1.3879730000	-2.2282520000
C	-2.7537190000	0.6955960000	-0.6056130000
Cl	-3.4581960000	1.8510420000	0.5335480000
Cl	-3.1780050000	1.1979840000	-2.2687830000
Cl	-7.1904670000	-0.5579730000	-0.8956770000
P	0.2419040000	0.9102810000	2.4973130000
F	-0.2396060000	2.2706670000	1.8143900000
F	-0.2535160000	1.3838820000	3.9397800000
F	-1.1916280000	0.2019320000	2.1980250000

F	0.7888740000	0.3493540000	1.0517800000
F	0.7743720000	-0.5172900000	3.1349030000
F	1.7259800000	1.5347820000	2.7692920000
K	3.3820490000	-0.4444370000	2.2922120000

Electronic Energy (Hartree)	Zero point correction (kcal/mol)	Electronic and Zero point correction (Hartree)	Gibbs free Energy (kcal/mol)	Electronic and Gibbs Free Energy (Hartree)	Number of Imaginary frequencies
-5254.746342	234.5410883	-5254.3726	142.1315293	-5254.519841	0

Figure 3S. Pre-reaction complex (case2)

F	1.0606370000	-0.3267680000	0.9775900000
P	-0.6235740000	-2.1122760000	-2.2511980000
F	-0.9271920000	-1.5632790000	-0.7307560000
F	-0.9138900000	-0.6234280000	-2.8191640000
F	0.9468110000	-1.6841690000	-2.0172200000
F	-0.2807460000	-3.5678320000	-1.6053510000
F	-0.2785370000	-2.6476590000	-3.7187640000
F	-2.1710250000	-2.5132090000	-2.4118980000
K	0.5394880000	0.9202600000	-0.9816190000
K	1.2161820000	-2.6265350000	0.5727530000
C	2.9833140000	3.0413070000	-1.5007570000
C	2.2375910000	3.7104990000	-0.5296980000
C	2.1738910000	3.2010400000	0.7618380000
C	2.8386200000	2.0216450000	1.0920170000
C	3.5748070000	1.3510890000	0.1213640000
C	3.6526630000	1.8683110000	-1.1770190000
H	3.0446230000	3.4305940000	-2.5098210000

H	1.7082770000	4.6215980000	-0.7820420000
H	1.5918300000	3.7101140000	1.5197570000
H	2.7548030000	1.6284860000	2.0941290000
H	4.2313920000	1.3568410000	-1.9383530000
C	4.3296890000	0.0711380000	0.4166360000
Cl	4.0660090000	-0.5737580000	2.0506610000
Cl	3.8652260000	-1.2058710000	-0.7696280000
Cl	6.0950860000	0.3585210000	0.2208840000
C	-1.9367240000	-0.4898870000	2.1582080000
H	-1.8891720000	0.3602530000	2.8412810000
N	-3.3148890000	-0.5401160000	1.6514660000
C	-3.6873700000	-0.8808160000	0.4226170000
C	-4.4408530000	-0.2542510000	2.3899010000
H	-3.0240530000	-1.1399110000	-0.3850940000
C	-5.5096570000	-0.4264160000	1.5705920000
H	-4.3766990000	0.0562170000	3.4193270000
H	-6.5658020000	-0.3017500000	1.7402030000
N	-5.0130090000	-0.8201670000	0.3474140000
C	-5.8062330000	-1.0909130000	-0.8492580000
H	-6.2878290000	-0.1705800000	-1.1757760000
H	-5.1394190000	-1.4569770000	-1.6269120000
H	-6.5547590000	-1.8459920000	-0.6164110000
H	-1.2624540000	-0.2952450000	1.3233010000
C	-1.5571210000	-1.7860020000	2.8666880000
H	-2.3326330000	-2.0513570000	3.5930740000
H	-1.5302350000	-2.5975220000	2.1278990000

C	-0.2065820000	-1.6516550000	3.5710670000
H	-0.3103490000	-0.9393820000	4.3959180000
H	0.5109930000	-1.2054380000	2.8744700000
C	0.2985330000	-2.9850550000	4.1171280000
H	0.3897910000	-3.7425160000	3.3303180000
H	1.2762690000	-2.8757410000	4.5914920000
H	-0.3926440000	-3.3896040000	4.8616830000
P	-2.1979900000	2.7194040000	-0.1288650000
F	-0.9341380000	2.4555860000	0.8664700000
F	-3.2207310000	2.2861760000	1.0429550000
F	-2.2623300000	4.2583300000	0.3414730000
F	-1.1368050000	3.1127730000	-1.3103060000
F	-3.4264860000	2.9486340000	-1.1472360000
F	-2.0884300000	1.1582710000	-0.6146530000

Electronic Energy (Hartree)	Zero point correction (kcal/mol)	Electronic and Zero point correction (Hartree)	Gibbs free Energy (kcal/mol)	Electronic and Gibbs Free Energy (Hartree)	Number of Imaginary frequencies
-5254.734776	233.596059	-5254.3625	140.3788952	-5254.511068	0

Figure 3S. Transition state (case2)

F	-1.7396880000	-1.1529550000	-0.1039460000
P	1.7014020000	-2.2926650000	1.8144290000
F	1.3088700000	-1.5459070000	0.3962110000
F	1.7648200000	-0.8391890000	2.5256100000
F	0.0907780000	-2.3508430000	2.1415370000
F	1.5566910000	-3.7215790000	1.0450500000
F	2.0275980000	-3.0308500000	3.1936380000
F	3.2576580000	-2.2107940000	1.4282800000
K	-0.5167510000	0.3130280000	1.4120260000

K	-0.7811870000	-3.3158540000	-0.2612880000
C	-3.4922850000	1.2700160000	2.6964290000
C	-2.9385970000	2.5063710000	2.3572200000
C	-2.7077750000	2.8304540000	1.0208910000
C	-3.0206970000	1.9237420000	0.0220520000
C	-3.5680680000	0.6763220000	0.3606540000
C	-3.8106190000	0.3552270000	1.7065480000
H	-3.6719170000	1.0196890000	3.7344890000
H	-2.6837750000	3.2156590000	3.1357350000
H	-2.2737870000	3.7865420000	0.7577480000
H	-2.8378040000	2.1847300000	-1.0116180000
H	-4.2277930000	-0.6048140000	1.9789400000
C	-3.7885110000	-0.3168410000	-0.6664440000
Cl	-3.2897660000	-0.0873470000	-2.2595450000
Cl	-4.4959030000	-1.8156160000	-0.3349420000
Cl	-6.2860320000	0.8450840000	-1.1545990000
C	1.8643140000	0.1389750000	-2.2128940000
H	1.5381000000	0.9182210000	-2.9022470000
N	3.2890760000	0.3697500000	-1.9482710000
C	3.9055380000	0.0908170000	-0.8044230000
C	4.2126160000	0.8676590000	-2.8389590000
H	3.4355470000	-0.3047370000	0.0809090000
C	5.4107730000	0.8855390000	-2.1987290000
H	3.9334110000	1.1665350000	-3.8354330000
H	6.3872460000	1.2041910000	-2.5223690000
N	5.1930730000	0.3939100000	-0.9306650000
C	6.2040570000	0.2519760000	0.1153830000
H	6.5649580000	1.2385260000	0.4011960000
H	5.7463320000	-0.2392330000	0.9709220000
H	7.0243200000	-0.3537840000	-0.2653320000
H	1.3427260000	0.2773170000	-1.2681780000
C	1.5998240000	-1.2524190000	-2.7695690000

H	2.1111950000	-1.3749400000	-3.7296750000
H	2.0134090000	-1.9945780000	-2.0780420000
C	0.0973430000	-1.4716430000	-2.9328540000
H	-0.3018390000	-0.7325440000	-3.6353690000
H	-0.4026390000	-1.2672290000	-1.9776540000
C	-0.2332360000	-2.8732780000	-3.4448840000
H	0.2637050000	-3.6535280000	-2.8558740000
H	-1.3108480000	-3.0613190000	-3.4435480000
H	0.1186910000	-3.0029500000	-4.4711250000
P	1.2196780000	2.9789040000	0.2781480000
F	-0.0020420000	2.2064430000	-0.4938260000
F	2.0805000000	2.9825200000	-1.0860890000
F	0.5631140000	4.3998220000	-0.0903620000
F	0.3372530000	2.9138390000	1.6537060000
F	2.4216380000	3.6956410000	1.0718750000
F	1.8340580000	1.5070940000	0.6636890000

Electronic Energy (Hartree)	Zero point correction (kcal/mol)	Electronic and Zero point correction (Hartree)	Gibbs free Energy (kcal/mol)	Electronic and Gibbs Free Energy (Hartree)	Number of Imaginary frequencies
-5254.694795	233.0909138	-5254.3233	141.51908	-5254.469271	1

Figure 3S. Post-reaction complex (case2)

F	-1.4681640000	0.9139060000	0.3556250000
P	1.0599810000	2.4114550000	-1.8552820000
F	1.0288340000	1.2651090000	-0.6858770000
F	1.2685200000	1.2342100000	-2.9529620000
F	-0.5774780000	2.2029270000	-1.9881090000
F	0.7646200000	3.5457820000	-0.7161580000
F	1.0171920000	3.5219640000	-3.0013800000
F	2.6368970000	2.5811280000	-1.6613610000
K	-0.3121560000	-0.6938110000	-1.9855260000
K	-1.7159480000	3.6438720000	-0.0203570000
C	-3.5191990000	-1.2515270000	-2.5162350000

C	-3.1527300000	-2.5918400000	-2.3909530000
C	-2.6417070000	-3.0612910000	-1.1864240000
C	-2.4844550000	-2.2000170000	-0.1034700000
C	-2.8462580000	-0.8627340000	-0.2323370000
C	-3.3653850000	-0.3854470000	-1.4412250000
H	-3.9267010000	-0.8799710000	-3.4484170000
H	-3.2728730000	-3.2670680000	-3.2295520000
H	-2.3548090000	-4.1000840000	-1.0817910000
H	-2.0822440000	-2.5815110000	0.8246220000
H	-3.6532130000	0.6541300000	-1.5505010000
C	-2.5269560000	0.1491790000	0.8367070000
Cl	-2.0003860000	-0.5130130000	2.3785390000
Cl	-3.8438650000	1.3261230000	1.1169920000
Cl	-5.6718460000	-1.9265460000	1.6937040000
C	1.5605810000	0.7280060000	2.3066700000
H	1.2562380000	0.3573420000	3.2860700000
N	2.8525890000	0.0975230000	2.0069310000
C	3.3976690000	0.0054500000	0.7972370000
C	3.6872830000	-0.5142830000	2.9139630000
H	2.9612060000	0.3692770000	-0.1173430000
C	4.7525900000	-0.9870860000	2.2161610000
H	3.4445710000	-0.5626330000	3.9620650000
H	5.6249900000	-1.5339180000	2.5317110000
N	4.5486560000	-0.6480900000	0.8987370000
C	5.3884320000	-1.0563620000	-0.2239710000
H	5.2506800000	-2.1225100000	-0.4018830000
H	5.0901920000	-0.4908650000	-1.1038960000
H	6.4283580000	-0.8453670000	0.0172820000
H	0.8459160000	0.3685000000	1.5658660000
C	1.6391760000	2.2480890000	2.2910200000
H	2.3796870000	2.5900680000	3.0203400000
H	1.9667280000	2.5814010000	1.3014250000

C	0.2708810000	2.8450700000	2.6130290000
H	-0.0150650000	2.5780340000	3.6348770000
H	-0.4730350000	2.3555810000	1.9718160000
C	0.2389780000	4.3635080000	2.4549630000
H	0.5544900000	4.6724140000	1.4529150000
H	-0.7527770000	4.7742320000	2.6694120000
H	0.9326810000	4.8327730000	3.1563500000
P	1.4901790000	-2.9357910000	-0.2070470000
F	0.5916260000	-1.6606860000	0.3175640000
F	2.5405550000	-2.6928460000	0.9878480000
F	0.6231690000	-3.9282270000	0.7134030000
F	0.4164800000	-3.1342310000	-1.4301340000
F	2.3700990000	-4.1554320000	-0.7742980000
F	2.3041060000	-1.8853630000	-1.1621780000

Electronic Energy (Hartree)	Zero point correction (kcal/mol)	Electronic and Zero point correction (Hartree)	Gibbs free Energy (kcal/mol)	Electronic and Gibbs Free Energy (Hartree)	Number of Imaginary frequencies
-5254.740943	234.9370468	-5254.3665	144.8291926	-5254.510143	0

Figure 4S. Pre-reaction complex

F	2.7528000000	-1.7635380000	0.9941770000
P	-2.4633050000	0.5849600000	1.8039370000
F	-1.9752650000	-0.9694820000	1.9537600000
F	-1.0389630000	1.0583080000	2.4491810000
F	-1.7694960000	0.6239380000	0.3250960000
F	-3.8513930000	0.0854930000	1.1498710000
F	-2.9078290000	2.1283130000	1.6334500000
F	-3.1152410000	0.5307290000	3.2781980000
K	0.6201480000	-0.6679410000	1.0930310000
C	1.1335660000	1.6352190000	-1.0400310000

C	0.8075460000	2.3043540000	0.1352340000
C	1.7235240000	2.3347380000	1.1838100000
C	2.9589820000	1.7095530000	1.0572650000
C	3.2870790000	1.0419090000	-0.1237150000
C	2.3712750000	1.0116160000	-1.1749790000
H	0.4258830000	1.5934430000	-1.8594840000
H	-0.1587310000	2.7813570000	0.2428030000
H	1.4766130000	2.8434980000	2.1078820000
H	3.6602260000	1.7515470000	1.8806170000
H	2.6124380000	0.5202080000	-2.1091430000
C	4.6967970000	0.5187570000	-0.3172410000
Cl	5.4630560000	-0.0385740000	1.1841370000
Cl	4.7946850000	-0.8122880000	-1.5158290000
Cl	5.7004250000	1.8742930000	-0.9729940000
C	-3.6792060000	0.1667470000	-2.4776050000
H	-4.2183260000	-0.0609800000	-3.3994830000
N	-3.7582880000	-1.0138000000	-1.6104790000
C	-2.7150190000	-1.6556660000	-1.0913900000
C	-4.9185020000	-1.6012990000	-1.1538960000
H	-1.6506680000	-1.4640020000	-1.2379680000
C	-4.5495860000	-2.6235330000	-0.3397160000
H	-5.8912660000	-1.2416720000	-1.4442430000
H	-5.1341110000	-3.3340760000	0.2199020000
N	-3.1730580000	-2.6352960000	-0.3174120000
C	-2.3161240000	-3.5549630000	0.4272550000
H	-2.5156000000	-3.4486460000	1.4919670000

H	-2.5199060000	-4.5755890000	0.1056580000
H	-1.2866190000	-3.2800790000	0.1981790000
H	-2.6246550000	0.3055230000	-2.7192750000
C	-4.2492740000	1.4063550000	-1.8010900000
H	-5.3001000000	1.2325750000	-1.5462950000
H	-3.7130980000	1.5797580000	-0.8658880000
C	-4.1339070000	2.6327450000	-2.7025470000
H	-4.6655980000	2.4487490000	-3.6421480000
H	-3.0813970000	2.7913700000	-2.9608410000
C	-4.6911280000	3.8824630000	-2.0275370000
H	-4.1615840000	4.0854070000	-1.0924090000
H	-4.5918750000	4.7604490000	-2.6695040000
H	-5.7511090000	3.7567790000	-1.7902090000
K	2.2955510000	-2.8420150000	-1.0485460000
F	0.0737470000	-1.9425930000	-0.9462180000

Electronic Energy (Hartree)	Zero point correction (kcal/mol)	Electronic and Zero point correction (Hartree)	Gibbs free Energy (kcal/mol)	Electronic and Gibbs Free Energy (Hartree)	Number of Imaginary frequencies
-4413.971164	222.0254113	-4413.6173	141.1168464	-4413.746281	0

Figure 4S. Transition state

F	2.9269350000	1.5131020000	-0.4118710000
P	-2.3814860000	-0.6412210000	-1.7991360000
F	-1.9999010000	0.9412860000	-1.9631900000
F	-0.9423580000	-1.0172120000	-2.4783580000
F	-1.6449540000	-0.6235090000	-0.3369610000
F	-3.7815720000	-0.2363960000	-1.1088320000

F	-2.7101980000	-2.2094450000	-1.6111490000
F	-3.0719470000	-0.6375670000	-3.2552380000
K	0.6354070000	0.7336910000	-1.1011540000
C	1.0689750000	-1.8519780000	0.9090940000
C	0.8324120000	-2.5118210000	-0.2951170000
C	1.7449540000	-2.3921340000	-1.3410410000
C	2.8812150000	-1.6079200000	-1.1946790000
C	3.1090230000	-0.9369030000	0.0107830000
C	2.2023770000	-1.0661800000	1.0676730000
H	0.3631790000	-1.9405290000	1.7260590000
H	-0.0613650000	-3.1107260000	-0.4206330000
H	1.5678390000	-2.9041750000	-2.2788490000
H	3.5850810000	-1.5264270000	-2.0127180000
H	2.3719720000	-0.5574030000	2.0076660000
C	4.2572630000	-0.0263630000	0.1345600000
Cl	5.2606330000	0.3674330000	-1.1850820000
Cl	4.6130820000	0.7973600000	1.6019490000
Cl	5.6995900000	-2.0370530000	0.8145750000
C	-3.4068010000	-0.1924600000	2.5061260000
H	-3.8860130000	0.0211430000	3.4638150000
N	-3.6885560000	0.9306750000	1.6048200000
C	-2.7715240000	1.6760450000	0.9934610000
C	-4.9407780000	1.3516080000	1.2124310000
H	-1.6861410000	1.6229540000	1.0730070000
C	-4.7580550000	2.3773250000	0.3416130000
H	-5.8395190000	0.8879030000	1.5824460000

H	-5.4636040000	2.9851380000	-0.1992740000
N	-3.3990410000	2.5587870000	0.2221700000
C	-2.7183050000	3.5428380000	-0.6161990000
H	-2.9668140000	3.3574090000	-1.6596300000
H	-3.0370850000	4.5428980000	-0.3248830000
H	-1.6490210000	3.4161880000	-0.4489130000
H	-2.3265920000	-0.2084990000	2.6565750000
C	-3.8950940000	-1.5165980000	1.9333630000
H	-4.9765530000	-1.4676630000	1.7686880000
H	-3.4253480000	-1.6785950000	0.9610640000
C	-3.5689460000	-2.6775840000	2.8695480000
H	-4.0418900000	-2.5075330000	3.8425520000
H	-2.4881700000	-2.7058380000	3.0448680000
C	-4.0308450000	-4.0132700000	2.2949710000
H	-3.5542070000	-4.2045740000	1.3294160000
H	-3.7841640000	-4.8411430000	2.9632330000
H	-5.1131780000	-4.0168140000	2.1394170000
K	2.1198940000	3.1363380000	1.2016040000
F	-0.0410130000	2.3140300000	0.6302250000

Electronic Energy (Hartree)	Zero point correction (kcal/mol)	Electronic and Zero point correction (Hartree)	Gibbs free Energy (kcal/mol)	Electronic and Gibbs Free Energy (Hartree)	Number of Imaginary frequencies
-4413.934992	220.9705678	-4413.5829	138.9331133	-4413.713589	1

Figure 4S. Post-reaction complex

F	3.3034640000	-1.5984610000	0.5942070000
P	-2.6858570000	0.5533190000	1.7721290000

F	-2.2080390000	-1.0080210000	1.9411460000
F	-1.4123780000	0.9920050000	2.7031860000
F	-1.7022000000	0.6573660000	0.4646350000
F	-3.9019250000	0.0781900000	0.8299170000
F	-3.0971760000	2.0981500000	1.5866000000
F	-3.6087420000	0.4229770000	3.0826350000
K	0.4326010000	-0.6203110000	1.5549410000
C	1.0058680000	1.9224930000	-0.3931840000
C	0.9590800000	2.5598560000	0.8434290000
C	1.9193270000	2.2673070000	1.8080900000
C	2.9170400000	1.3337930000	1.5429060000
C	2.9576330000	0.6919360000	0.3035040000
C	1.9963120000	0.9851730000	-0.6643540000
H	0.2648260000	2.1484900000	-1.1496720000
H	0.1780000000	3.2806780000	1.0533390000
H	1.8958750000	2.7626850000	2.7709380000
H	3.6558630000	1.1166430000	2.3036460000
H	2.0120680000	0.4981020000	-1.6297190000
C	3.9157680000	-0.4560530000	0.0985730000
Cl	5.4425720000	-0.3045760000	0.9728030000
Cl	4.2604010000	-0.8172080000	-1.6132040000
Cl	5.3747680000	2.8361320000	-1.3369260000
C	-2.9619420000	0.2886140000	-2.6659200000
H	-3.2946930000	0.0972730000	-3.6881080000
N	-3.2355750000	-0.9181210000	-1.8755830000
C	-2.3356400000	-1.6068520000	-1.1786220000

C	-4.4752330000	-1.4897150000	-1.6905820000
H	-1.2651750000	-1.4332200000	-1.1034470000
C	-4.3032670000	-2.5483530000	-0.8579540000
H	-5.3598890000	-1.0935320000	-2.1598770000
H	-5.0052290000	-3.2595260000	-0.4563730000
N	-2.9622960000	-2.5991120000	-0.5528670000
C	-2.3061670000	-3.5679370000	0.3218920000
H	-2.7260150000	-3.4873250000	1.3229310000
H	-2.4633440000	-4.5708650000	-0.0729810000
H	-1.2446120000	-3.3233260000	0.3308840000
H	-1.8792010000	0.4179130000	-2.6736700000
C	-3.6537420000	1.5157380000	-2.0862990000
H	-4.7345380000	1.3425930000	-2.0510670000
H	-3.3148720000	1.6660060000	-1.0593160000
C	-3.3602970000	2.7622330000	-2.9174740000
H	-3.7088410000	2.6087420000	-3.9441570000
H	-2.2770720000	2.9141170000	-2.9711190000
C	-4.0231680000	4.0019770000	-2.3244670000
H	-3.6734700000	4.1761230000	-1.3029830000
H	-3.7999240000	4.8935940000	-2.9142710000
H	-5.1097010000	3.8838380000	-2.2899000000
K	2.1775950000	-3.3798500000	-1.1538650000
F	0.4156810000	-2.0222360000	-0.3957670000

Electronic Energy (Hartree)	Zero point correction (kcal/mol)	Electronic and Zero point correction (Hartree)	Gibbs free Energy (kcal/mol)	Electronic and Gibbs Free Energy (Hartree)	Number of Imaginary frequencies
-4413.986591	222.6008375	-4413.6319	141.0101698	-4413.761877	0

Figure 5S. Pre-reaction complex

F	-1.4322760000	-0.2909680000	0.4005470000
P	1.8173840000	3.1157950000	0.4774510000
F	1.2055790000	3.2513590000	-1.0430600000
F	0.3704010000	3.5647190000	1.0629020000
F	1.2994190000	1.5561530000	0.4763540000
F	3.2192950000	2.6066300000	-0.1703230000
F	2.3934550000	2.9334140000	1.9641680000
F	2.3095320000	4.6400770000	0.4252890000
K	-1.2356550000	1.9269050000	-0.5374230000
C	-4.0053530000	3.0414470000	0.5892090000
C	-4.0254530000	3.3660970000	-0.7649010000
C	-4.1825860000	2.3556250000	-1.7102400000
C	-4.3279730000	1.0319060000	-1.3074080000
C	-4.3083850000	0.7084700000	0.0504230000
C	-4.1494460000	1.7189570000	0.9985310000
H	-3.8806870000	3.8172550000	1.3351880000
H	-3.9178720000	4.3969540000	-1.0808750000
H	-4.1972050000	2.5933180000	-2.7670600000
H	-4.4719370000	0.2643290000	-2.0581190000
H	-4.1404630000	1.4900610000	2.0557770000
C	-4.6307170000	-0.7071010000	0.4899680000
Cl	-4.0132000000	-1.9485560000	-0.6501230000
Cl	-4.0262410000	-1.1051400000	2.1118500000
Cl	-6.4283900000	-0.8802040000	0.5184500000
C	2.7876310000	-0.2889730000	2.6734070000

H	3.2725960000	-0.3829670000	3.6466370000
N	1.8908370000	-1.4447470000	2.5369630000
C	0.7061900000	-1.4571640000	1.9295190000
C	2.1974370000	-2.7246790000	2.9456310000
H	0.1725890000	-0.6337980000	1.4610290000
C	1.1629430000	-3.5194710000	2.5732140000
H	3.1175270000	-2.9479690000	3.4594420000
H	0.9923080000	-4.5750830000	2.7009870000
N	0.2481500000	-2.7077960000	1.9400190000
C	-1.0320020000	-3.1299060000	1.3724020000
H	-1.6236700000	-3.6159920000	2.1474440000
H	-1.5321070000	-2.2267850000	1.0182590000
H	-0.8458590000	-3.8298630000	0.5574060000
H	2.1697470000	0.6079390000	2.6773000000
C	3.8170700000	-0.2470910000	1.5497350000
H	4.2575660000	-1.2418050000	1.4286230000
H	3.3043010000	-0.0061600000	0.6153610000
C	4.9207450000	0.7728580000	1.8144010000
H	5.4592240000	0.4926530000	2.7260890000
H	4.4768610000	1.7553090000	1.9937230000
C	5.8938720000	0.8527210000	0.6418240000
H	5.3737980000	1.1591480000	-0.2697630000
H	6.6907390000	1.5751990000	0.8324190000
H	6.3575160000	-0.1197610000	0.4524300000
K	-0.9417040000	-1.3348920000	-1.7384610000
F	-0.2740360000	0.8623620000	-2.5271380000

P	2.2992540000	-2.3684730000	-1.4248010000
F	1.6338710000	-1.7951470000	-2.8129380000
F	3.0064290000	-3.5545010000	-2.2342970000
F	3.5731940000	-1.3873160000	-1.6681990000
F	1.5458720000	-1.1288440000	-0.6485440000
F	2.9127860000	-2.8790640000	-0.0356480000
F	0.9639520000	-3.2711340000	-1.1930290000
K	2.1091790000	0.9632750000	-2.1853230000

Electronic Energy (Hartree)	Zero point correction (kcal/mol)	Electronic and Zero point correction (Hartree)	Gibbs free Energy (kcal/mol)	Electronic and Gibbs Free Energy (Hartree)	Number of Imaginary frequencies
-5954.63017	236.0565237	-5954.254	143.8157648	-5954.400985	0

Figure 5S. Transition state

F	-1.4922110000	-0.8111380000	-0.5676340000
P	0.8719840000	3.1893400000	0.4478080000
F	0.7358240000	3.2846800000	-1.1898140000
F	-0.7474570000	3.2879600000	0.5512940000
F	0.7254600000	1.5560560000	0.2926150000
F	2.4770360000	3.0194770000	0.2686350000
F	0.9916740000	3.0372360000	2.0395910000
F	1.0127590000	4.7811160000	0.5402240000
K	-1.3801200000	1.5143180000	-1.4626870000
C	-3.8641020000	2.6630310000	0.4304990000
C	-4.4013370000	2.9312060000	-0.8294950000
C	-4.7160150000	1.8871110000	-1.7028790000
C	-4.4960150000	0.5755490000	-1.3227620000
C	-3.9480250000	0.2999330000	-0.0547790000
C	-3.6369820000	1.3558880000	0.8234340000
H	-3.6187240000	3.4748740000	1.1027070000
H	-4.5769390000	3.9568200000	-1.1318550000

H	-5.1398400000	2.0996940000	-2.6760960000
H	-4.7551890000	-0.2274020000	-1.9997730000
H	-3.2186370000	1.1594210000	1.8017610000
C	-3.6597440000	-1.0541800000	0.3213730000
Cl	-3.9488800000	-2.3545650000	-0.7029680000
Cl	-3.0549000000	-1.4491840000	1.8382360000
Cl	-6.4327240000	-1.1929890000	1.3426870000
C	1.8718760000	0.0260560000	2.9268690000
H	2.0678630000	0.0071890000	4.0002340000
N	1.3295390000	-1.2946760000	2.5769630000
C	0.4405840000	-1.5545710000	1.6214380000
C	1.7529620000	-2.4839000000	3.1276970000
H	-0.0720810000	-0.8460910000	0.9801550000
C	1.0925700000	-3.4788800000	2.4841800000
H	2.4801250000	-2.5101810000	3.9220980000
H	1.1211670000	-4.5485740000	2.6046340000
N	0.2828560000	-2.8760450000	1.5480780000
C	-0.6032200000	-3.5703440000	0.6162940000
H	-1.3194210000	-4.1698500000	1.1775830000
H	-1.1235620000	-2.8064210000	0.0396710000
H	-0.0069620000	-4.2143520000	-0.0287960000
H	1.0961600000	0.7638040000	2.7273360000
C	3.1429020000	0.3260120000	2.1401460000
H	3.8009530000	-0.5479050000	2.1766160000
H	2.8754680000	0.4830660000	1.0921720000
C	3.8845410000	1.5462900000	2.6778800000
H	4.1954050000	1.3512470000	3.7097980000
H	3.2101940000	2.4057550000	2.7034580000
C	5.1047550000	1.8733030000	1.8220080000
H	4.8049450000	2.1056900000	0.7966090000
H	5.6484920000	2.7351050000	2.2155820000
H	5.7951450000	1.0255850000	1.7863240000

K	-0.0721090000	-1.6501380000	-2.4232950000
F	0.3220520000	0.6705220000	-2.9745320000
P	3.0440500000	-1.9717140000	-1.0729140000
F	2.7600690000	-1.5027920000	-2.6199960000
F	4.2066350000	-2.9545210000	-1.5617280000
F	4.0891120000	-0.7368800000	-0.9217400000
F	1.8321540000	-0.9453620000	-0.6296580000
F	3.2574600000	-2.3901230000	0.4587690000
F	1.9235250000	-3.1408600000	-1.2561620000
K	2.4255650000	1.2792080000	-1.9553000000

Electronic Energy (Hartree)	Zero point correction (kcal/mol)	Electronic and Zero point correction (Hartree)	Gibbs free Energy (kcal/mol)	Electronic and Gibbs Free Energy (Hartree)	Number of Imaginary frequencies
-5954.588956	234.7130259	-5954.2149	138.9588412	-5954.367511	1

Figure 5S. Post-reaction complex

F	-1.5482230000	-1.0260860000	0.1723940000
P	0.8731670000	3.2024130000	0.4080450000
F	0.5441180000	3.4995850000	-1.1856120000
F	-0.6569820000	3.6114970000	0.7562980000
F	0.3633980000	1.6620020000	0.1431440000
F	2.3676360000	2.7461900000	-0.0343810000
F	1.1732070000	2.8471980000	1.9416130000
F	1.3592590000	4.7136760000	0.6029920000
K	-1.8852350000	2.2890110000	-1.3856370000
C	-3.3386530000	2.4654790000	1.4575680000
C	-4.3992870000	2.8863230000	0.6539450000
C	-4.9938280000	1.9977980000	-0.2355760000
C	-4.5399350000	0.6823540000	-0.3291770000
C	-3.4758310000	0.2713200000	0.4676210000
C	-2.8696380000	1.1623450000	1.3620490000
H	-2.8697210000	3.1539410000	2.1486250000
H	-4.7631980000	3.9041160000	0.7288820000

H	-5.8252940000	2.3161220000	-0.8524470000
H	-5.0368360000	-0.0110450000	-0.9924900000
H	-2.0426710000	0.8388710000	1.9850160000
C	-2.9030730000	-1.1202130000	0.4074060000
Cl	-3.5410540000	-2.1286490000	-0.9094770000
Cl	-3.0800250000	-1.9812110000	1.9530820000
Cl	-7.0210020000	-1.5424650000	0.5314770000
C	2.8191430000	-0.0452700000	2.8956520000
H	3.3652390000	-0.0402310000	3.8398240000
N	2.3066890000	-1.4107360000	2.7127730000
C	1.1972560000	-1.7460280000	2.0621800000
C	2.9769680000	-2.5598810000	3.0652190000
H	0.4838840000	-1.0578920000	1.6382350000
C	2.2368530000	-3.6051910000	2.6157810000
H	3.9118820000	-2.5270150000	3.5990010000
H	2.3926290000	-4.6688260000	2.6801010000
N	1.1334890000	-3.0725260000	1.9895270000
C	0.0760170000	-3.8472840000	1.3456500000
H	-0.5068240000	-4.3690400000	2.1035140000
H	-0.5638070000	-3.1616830000	0.7976960000
H	0.5311790000	-4.5593130000	0.6603080000
H	1.9615120000	0.6215980000	2.9853210000
C	3.7163140000	0.3563120000	1.7316600000
H	4.4605140000	-0.4302810000	1.5674870000
H	3.1090560000	0.4210470000	0.8239420000
C	4.4202860000	1.6858310000	1.9857780000
H	5.0066960000	1.6118100000	2.9079170000
H	3.6739390000	2.4693350000	2.1424550000
C	5.3342370000	2.0640840000	0.8240820000
H	4.7668400000	2.1338420000	-0.1071920000
H	5.8181110000	3.0281680000	0.9963230000
H	6.1173810000	1.3133460000	0.6851860000

K	-0.6172440000	-1.6855850000	-2.4210740000
F	-0.5722780000	0.6942180000	-2.6136630000
P	2.6401370000	-1.9072880000	-1.4471300000
F	2.0087170000	-1.4048510000	-2.8898490000
F	3.7194010000	-2.7985040000	-2.2172980000
F	3.6213420000	-0.6144800000	-1.4762440000
F	1.4909890000	-0.9744940000	-0.7214730000
F	3.2024020000	-2.3549800000	-0.0181690000
F	1.5673570000	-3.1309470000	-1.4525660000
K	1.7572160000	1.2881810000	-2.2652700000

Electronic Energy (Hartree)	Zero point correction (kcal/mol)	Electronic and Zero point correction (Hartree)	Gibbs free Energy (kcal/mol)	Electronic and Gibbs Free Energy (Hartree)	Number of Imaginary frequencies
-5954.636757	236.3250978	-5954.2601	141.2818814	-5954.411609	0
